# Superior Performances of B-doped LiNi_0.84_Co_0.10_Mn_0.06_O_2_ cathode for advanced LIBs

**DOI:** 10.1038/s41598-019-54115-z

**Published:** 2019-11-26

**Authors:** Seung-Hwan Lee, Bong-Soo Jin, Hyun-Soo Kim

**Affiliations:** Next-generation Battery Research Center, Korea Electrotechnology Research Institute, Changwon, 641-120 South Korea

**Keywords:** Batteries, Batteries

## Abstract

Boron-doped Ni-rich LiNi_0.84_Co_0.10_Mn_0.06_O_2_ (B-NCM) cathode material is prepared and its electrochemical performances are investigated. The structural properties indicate that the incorporation of boron leads to highly-ordered layered structure and low cation disordering. All samples have high areal loadings of active materials (approximately 14.6 mg/cm^2^) that meets the requirement for commercialization. Among them, the 1.0 wt% boron-doped NCM (1.0B-NCM) shows the best electrochemical performances. The 1.0B-NCM delivers a discharge capacity of 205. 3 mAh g^−1^, cyclability of 93.1% after 50 cycles at 0.5 C and rate capability of 87.5% at 2 C. As a result, we can conclude that the 1.0B-NCM cathode can be regarded as a promising candidate for the next-generation lithium ion batteries.

## Introduction

Recently, lithium-ion batteries (LIBs) are most commonly used in electric vehicles (EVs) and hybrid electric vehicles (HEVs) and portable devices due to superior energy density although they have suffered from inferior cycle life and rate capability. In order to overcome these shortcomings, there have been many attempts for safe and high-rate LIBs^1–3^. However, much more research efforts are still needed for next-generation LIBs.

It has been reported that the cathode plays an important role in determining not only electrochemical performances, but also safety and reliability^4^. Among the various cathode materials, the Li(Ni,Co,Mn)O_2_ (NCM) has been regarded as a strong candidate to replace expensive and toxic LiCoO_2_ cathode, which is the most common cathode material in commercial LIBs^5^. For NCM cathode, the key point for increasing the discharge capacity has been a steady increase of the Ni contents. However, the long-term cycling stability of Ni-rich NCM (Li(Ni_x_Co_y_Mn_1-x−y_)O_2_ (x > 0.8)) is deteriorated proportionally with increasing Ni contents. It can be explained by the reduction reaction of Ni^4+^ and oxygen release, resulting in destroy the crystal structure^6^. The more Ni contents, the lower the onset temperature of the phase transition, accompanied by the oxygen release. This phenomenon causes a decrease in electrochemical performance.

Many approaches, such as coatings^7^, substitution^7,8^, electrolyte additives and single crystal^9,10^, have been designed to improve the electrochemical performance of Ni-rich NCM cathode. Among them, the substitution is one of the effective way to stabilize the crystal structure, and deliver the long cycle life and high rate capability without capacity fading. It can be explained by the alleviating cation migration from the transition‐metal site to lithium site, maintaining the electrode stability from volume contraction or expansion. The excellent electrochemical performances of lithium-rich manganese-based oxide could be obtained based on structure stability, resulting from polyanion doping of (BO_3_)^3−^ and (BO_4_)^5−^ ^11,12^.

Therefore, in this paper, we propose to employ the B^3+^ as a substituent to suppress the migration of transition-meal ions and stabilize the crystal structure of the Ni-rich NCM. The results indicate that appropriate boron content can significantly enhance the electrochemical performances.

## Experimental

For the synthesis of B-NCM powders, the Ni_0.84_Co_0.10_Mn_0.06_(OH)_2_ precursor was prepared using NiSO_4_·6H_2_O, CoSO_4_·7H_2_O and MnSO_4_·H_2_O by co-precipitation method. The NaOH and NH_4_OH solution were also used as a chelating agent. The LiOH·H_2_O was mixed with as-prepared spherical Ni_0.84_Co_0.10_Mn_0.06_(OH)_2_ precursor in a molar ratio 1.05:1 and BH_3_O_3_ as a boron source with molar ratio of 0, 0.5, 1.0 and 1.5 wt%. The mixture was calcined at 500 °C for 5 h and then sintered at 760 °C for 15 h in air.

The cathodes were prepared by mixing 96 wt% NCM powder, 2 wt% conductive carbon black binder and 2 wt% polyvinylidene fluoride. After that, the *N*-Methyl pyrrolidinone (NMP) solvent was added. The mixed slurry was casted on Al foil and then dried at 120 °C for over 12 h to remove the NMP solvent. The 2032 coin cells were assembled with Li metal disc as an anode, 1 M LiPF_6_ in ethylene carbonate, dimethyl carbonate, and ethyl methyl carbonate (EC:DMC:EMC 1:1:1, v/v/v/) as electrolyte and polyethylene (PE, 20 μm in thickness) was used as a separator. All coin cells were assembled in argon-gas-filled glove box.

The morphology and crystal structure of the pristine and B-doped NCM powders were measured using X-ray diffraction (XRD, Philips, X-pert PRO MPD), field emission scanning electron microscope (FE-SEM, Hitachi S-4800) and surface area was measured by N_2_ adsorption measurement on a BELSORP-mini instrument. The lattice parameters were determined using Rietveld refinements with High Score Plus. The sheet resistance coated on Al film were measured by Keithly 617 Multimeter (using the Ohms function as a current source) and a Agilent 34401 A multimeter.

The electrochemical performances were measured using an electrochemical equipment (TOSCAT-3100, Toyo system). The cyclic voltammetry (CV) were measured by multi potentiostat (VSP300, Bio-Logic) between 3.0 and 4.5 V at a scan rate of 0.1 mV s^−1^. The electrochemical impedance spectra (EIS) was obtained after first cycle using the electrochemical interface and a frequency response analyzer (Bio-Logic, VSP-300). The amplitude of the AC signal was 10 mV over a frequency range from 1 MHz to 10 mHz.

## Results and Discussion

**Figure 1:**
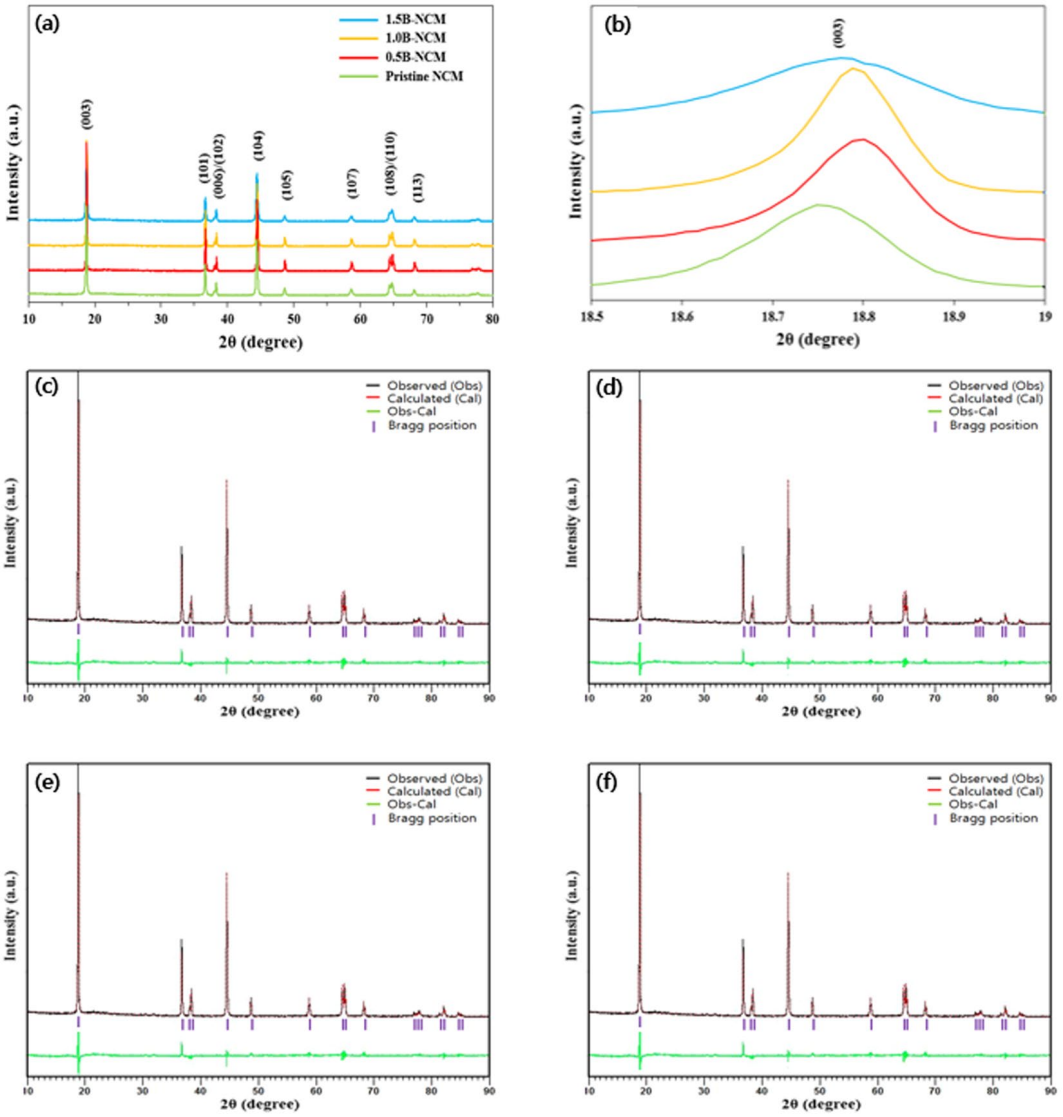
(**a**) XRD patterns of pristine and B-doped NCM and (**b**) magnified views of the (003) peaks; Rietveld refinement of (**c**) pristine, (**d**) 0.5B, (**e**) 1.0B and (**f**) 1.5B-doped NCM.

Figure 1(a) shows the XRD patterns recorded for pristine and B-doped NCM powders. There is no significant peak change as the concentration of B increases. All diffraction peaks of the sample can be well indexed on the basis of a layered structure of hexagonal α-NaFeO_2_ with a space group of R$\bar{3}$m^13,14^. No extra diffraction peaks were observed. It means that the absence of impurity in all samples. It is obvious that there are the clear splitting of (006)/(102) and (108)/(110) peaks regardless of doping concentration, indicating 2-D order level of the layered structure^15^.

Among them, the 1.0B-doped NCM exhibits the largest I_(003)_/I_(104)_ value of 1.62, as shown in Table 1. It demonstrates that 1.0B-doped NCM has an orderly layered structure with desirable cation ordering^2^. It can be explained by the incorporation of B into the layered structure, leading to high driving force to suppress Ni ions movement to toward Li site. Moreover, the smallest in full-width at half maximum (FWHM) value of 1.0B-doped NCM indicates that it has the highest crystallinity compared to others, as shown in Fig. (b)^16^. To further demonstrate the crystal structure, the XRD date is analyzed via Rietveld refinement. Figure 1(c–f) shows the Rietveld refinement result for the pristine and B-doped NCM powders. The calculated Chi values are summarized in Table 2. Also, we can confirm that the distance between two adjacent lattice increase with increasing B substitution^17^.

**Table 1:** I_003_/I_104_ values of pristine and B-doped NCM.

Sample	Pristine	B0.5-NCM	B1.0-NCM	B1.5-NCM
I_003_/I_104_	1.52	1.57	1.62	1.45

**Table 2:** Results of structural analysis obtained from X-ray Rietveld refinement of the pristine and B-doped NCM.

Sample	a-axis [Å]	c-axis [Å]	chi-square	d(003) (Å)
Pristine	2.8721	14.1948	3.58	4.7258
B0.5-NCM	2.8739	14.1975	3.63	4.7272
B1.0-NCM	2.8748	14.1989	3.76	4.7298
B1.5-NCM	2.8754	14.2001	3.89	4.7311

Table 2 shows the lattice parameters of the pristine and B-doped NCM. The lattice parameters increase sharply by B substitution compared to pristine NCM while the lattice parameters increase slightly with additional B concentration. This phenomenon can be explained by the dual-site occupation of octahedral sites and tetrahedral interstices of the oxygen array in the transition metal and lithium layers. Li *et al*. reported that B^3+^ can mostly occupy the tetrahedral interstitial of the packed oxygen in the transition metal and lithium layer, leading to increase in lattice parameters remarkably^18,19^. It enables the fast and smooth lithium ion kinetics. However, the excess B^3+^ tends to occupy the vacancies in transition metals except that a small amount of B^3+^ occupies the tetrahedral interstice. Therefore, the high amount B doped NCM has slightly increased lattice parameters compared to those of low amount B doped sample since the volume of octahedral interstice of transition metal layer is much larger compared to that of B^3+ ^^12^.

**Figure 2:**
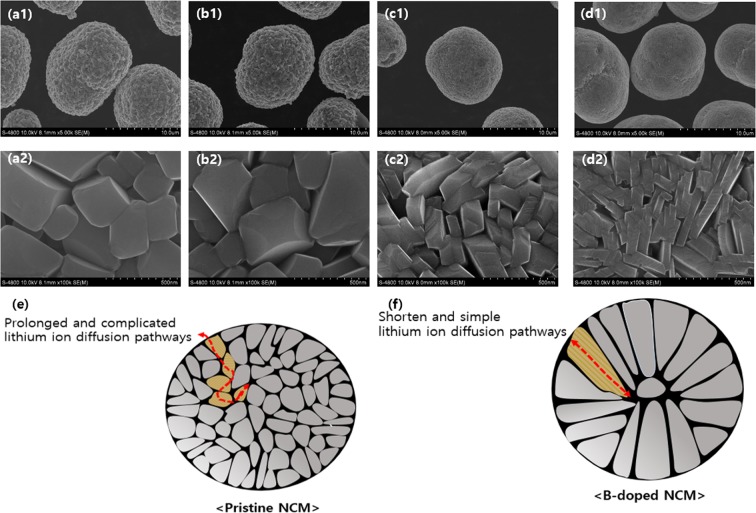
FESEM images of (**a**) pristine, (**b**) 0.5B, (**c**) 1.0B and (**d**) 1.5B-doped NCM; Schematic illustration of (**e**) pristine and (**f**) B-doped NCM.

The particulate morphology of the pristine and B-doped NCM after sintering process were observed by SEM images, as shown in Fig. 2(a). All B-doped samples have spherical shapes with average diameter of approximately 12 µm, identical to that of pristine and no significant changes in the secondary particle size. However, the primary particle size, composing of secondary particle, is affected by the B doping (inset of Fig. 2(a)) and it decreases in proportional to the doping concentration. The pristine NCM has relatively rounded and equiaxed shape while the introduction of B into NCM shows more rectangular and stretched shape. Park *et al*. reported that the B doping has a strong influence on the microstructure^20^. The primary particle elongated in the radial direction by B substitution since it plays roles in crystal growth flux and sintering agent, as shown in Fig. 2(b). Based on structural properties, the B-doped NCM is expected to deliver better electrochemical performances.

**Figure 3:**
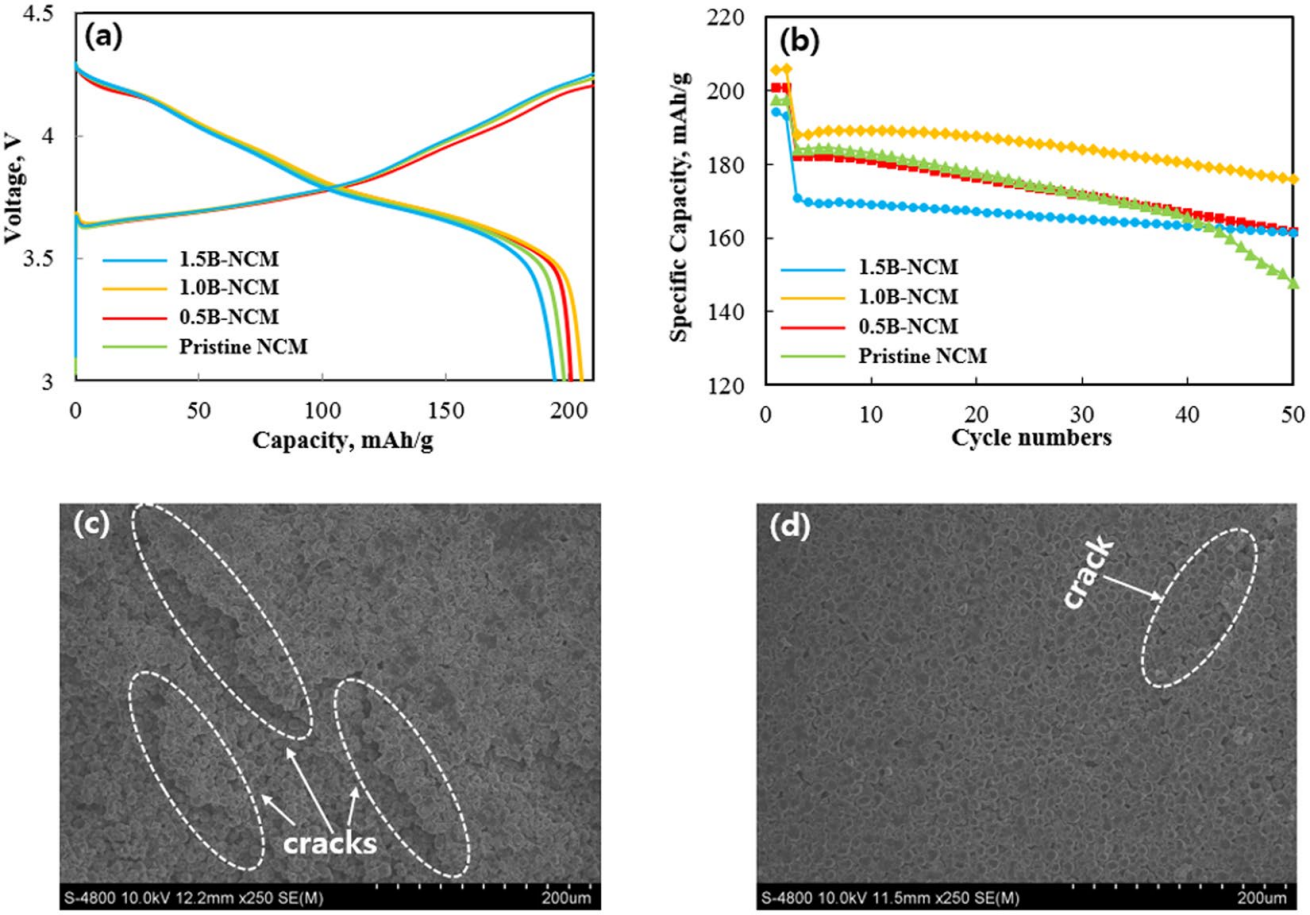
(**a**) Initial charge-discharge curves, (**b**) cyclability of pristine and B-doped NCM. SEM image of (**c**) pristine and (**d**) 1.0B-doped NCM.

For electrochemical tests, the pristine and B-doped NCM cathodes with very high areal loadings of active materials (approximately 14.6 mg/cm^2^) are prepared to meet similar condition of commercial cathode. The initial charge-discharge curves of pristine and B-doped NCM were measured in the voltage range of 3.0–4.3 V, as shown in Fig. 3(a). We can confirm that all curves are smoother without polarization, and gradually decreasing discharge voltage from 4.5 to 3.0 V without any additional plateau. It means that B introduction did not influence the electrochemical behaviour of NCM^3^. There are no obvious differences in discharge capacities for all samples. The discharge capacity increased as the B content increased up to 1 wt%. However, the discharge capacity decays when the excess B content is more than 1 wt%. The initial discharge capacity of pristine, 0.5, 1.0 and 1.5 wt% B-doped NCM are 198.3, 201.4, 205.3 and 194.7 mAh g^−1^, respectively.

Figure 3(b) shows the cycling stability of pristine and B-doped NCM at a rate of 0.5 C. It can be seen that capacity retention of pristine NCM is only 81.4% after 50 cycles while B-doped NCM delivers higher cyclability compared to pristine under the same condition. Especially, the 1.0B-doped NCM has the highest long-term cyclic stability (93.1%) among B-doped samples. The poor cycle life of pristine NCM can be explained by spherical secondary particles composed of closely packed primary particles with random orientations^21^. It provides the long pathway for lithium ion diffusion inside the secondary particles since lithium ions are required to transport across the grain boundary^22^. Also, Ni-rich NCM cathode with Ni contents of over than 80%, the rapid capacity fading was occurred due to micro-cracks, originated from anisotropic volume change. Moreover, the electrolyte infiltration to the cracks can block lithium-ion movement by forming the solid electrolyte interface (SEI) layer on the surface of the primary particles^23^. Ultimately, crack formation and propagation along the grain boundaries until collapse of secondary particle, resulting in performance degradation upon cycling. On the contrary, the reason for the stable cyclability of B-doped NCM may be that radially aligned primary particle maintain the mechanical integrity, beneficial to low internal strain^22^. We can confirm that the B addition results in significant improvement of cycle stability. However, the further increase of B causes accelerated performance decay due to blocking lithium ion diffusion pathways by crystal structure distortion^11,24^. Figure 3(c,d) indicates the SEM images of pristine and 1.0B-doped NCM after cycle test. The pristine NCM has multiple cracks on the surface, closely related to the stress derived from shrinkage and expansion of NCM. However, we can confirm that 1.0B-doped NCM has sufficient buffer space to suppress the structural degradation. Therefore, it maintains stable with relatively less cracks, regarded to have a negative effect on the electrochemical performances.

**Figure 4:**
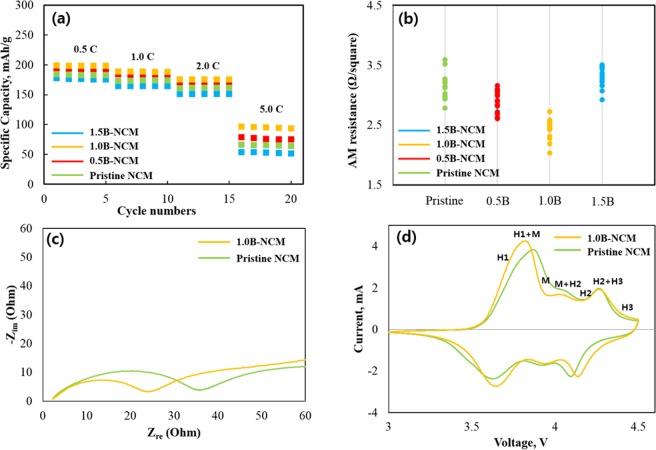
(**a**) Rate capability and (**b**) sheet resistance of pristine and B-doped NC. (**c**) Nyquist plots and (**d**) CV curves of pristine and 1.0B-doped NCM.

Figure 4(a) illustratesthe rate capability of pristine and 1.0B-doped samples at the rate of 0.5, 1.0, 2.0 and 5.0 C. The five cycles are tested for each current density. It is clear that discharge capacity of all sample quickly decreased with increasing current density. Among them, pristine NCM shows an abrupt capacity drop as the current density is increased, especially at 5.0 C compared to others. On the other hand, the 1.0B-doped NCM preserves the capacity well even at the high current density, and exhibits the higher discharge capacity compared to pristine NCM under the same condition. It indicates that low sheet resistance (Fig. 4(b)) and enlarged crystal lattice of 1.0B-doped NCM can increase the delivery of lithium ions and then contribute to large capacity. The 1.0B-doped NCM shows the minimum resistivity value of 2.43 Ω/square, which is a significant improvement over pristine NCM, indicating that B introduction enhance the conducting ability of pristine NCM. After the substitution of B, the metal-oxygen bonds are lengthened owing to the strong B-O bond, leading to the local octahedron geometrical distortion. Also the valence of the transition metal of NCM decreases. The local structural change can cause the variation of electronic structure, resulting in higher electronic conductivity^25,26^. Moreover, an excellent rate performance of 1.0B-doped NCM is related to the high BET surface area of 1.01 m^2^ g^−1^ than that of pristine NCM (0.88 m^2^ g^−1^).

To better understand the underlying electrochemical performances, impedance spectra is measured. Figure 4(c) shows the Nyquist plot of the pristine and 1.0B-doped NCM. There is no significant difference in electrolyte resistance (R_e_) between both samples since the electrolyte for both samples is the same. The 1.0B-doped sample has much smaller charge-transfer resistance (R_ct_) of 24.6 Ω in comparison with pristine sample (35.1 Ω). It can explained by the lower cation mixing, derived from B incorporation. In addition, it was reported that the surface of both sample have a similar degree of surface degradation by side reaction with electrolyte, thus the increase in resistance can be explained by the electrolyte penetration into the inside of the secondary particle^20^. Furthermore, the primary particles of B-doped NCM are aligned from surface to core, facilitating fast lithium ions transfer inside the secondary particles without crossing grain boundaries, as mentioned above.

Figure 4(d) shows the CV curves of pristine and 1.0B-doped NCM to confirm the electrochemical behaviour and phase transition. Both curves have several redox peaks, associated with the phase transition, consistent with previous studies^6,20^. The shape of the CV curves does not change drastically regardless of B incorporation. The oxidation and reduction peaks correspond to the delithiation and lithiation process, respectively. Both samples experience multiple phase transitions during delithiation: the original hexagonal structure (H1) changed into monoclinic (M) and then two other hexagonal structure (H2, H3). Among all phase transitions, transition from H2 to H3 around 4.26 V leads to abrupt lattice shrinkage along c-direction, leading to performance decay^6^. The redox peaks of pristine and 1.0B-doped NCM are found around 3.87/3.82 V and 3.63/3.67 V, corresponding to Ni^2+^/Ni^4+^. Also, the redox peaks were found around 4.25/4.24 V and 4.14/4.08 V, corresponding to Co^3+^/Co^4+^. The potential difference (polarization) between oxidation and reduction peak of 1.0B-doped NCM is smaller (0.15 V) than pristine NCM (0.24 V), demonstrating that B incorporation is favorable for lithium ion movement and reversibility, resulting in better electrochemical activity^26–28^.

## Conclusion

In this work, we have successfully prepared B-doped Ni-rich NCM materials. It is worthy to mention than an appropriate amount of B substitution can significantly improve the electrochemical performances. We demonstrate that the 1.0B-doped NCM offers better performances compared to pristine sample. Such enhancements are due to introduction of boron into LiNi_0.84_Co_0.10_Mn_0.06_O_2_, which effectively suppress the structural degradation derived from anisotropic volume change during lithium insertion/extraction and cation intermixing. These findings indicate that 1.0B-doped NCM cathode could be an effective strategy for advanced lithium ion batteries.
